# A statistical model to assess the risk of communicable diseases associated with multiple exposures in healthcare settings

**DOI:** 10.1186/1471-2288-13-26

**Published:** 2013-02-20

**Authors:** Cécile Payet, Nicolas Voirin, Philippe Vanhems, René Ecochard

**Affiliations:** 1 Hospices Civils de Lyon, Service d’Hygiène, Epidémiologie et Prévention, Unité Epidémiologie et Biomarqueurs de l’Infection, F-69437, Lyon, France; 2 CNRS, UMR5558, Laboratoire de Biométrie et Biologie Evolutive, Equipe Epidémiologie et Santé Publique, Université de Lyon; Université Lyon 1, F-69100, Villeurbanne, France; 3 Hospices Civils de Lyon, Service de Biostatistique, F-69003, Lyon, France

**Keywords:** Disease transmission, Infectious, Health facilities, Influenza, Human, Models, Statistical, Risk

## Abstract

**Background:**

The occurrence of communicable diseases (CD) depends on exposure to contagious persons. The effects of exposure to CD are delayed in time and contagious persons remain contagious for several days during which their contagiousness varies. Moreover when multiple exposures occur, it is difficult to know which exposure is associated with the CD.

**Methods:**

A statistical model at the individual level is presented to estimate the risk of CD to patients, in healthcare settings, with multiple observed exposures to other patients and healthcare workers and unobserved exposures to unobserved or unobservable sources. The model explores the delayed effect of observed exposure, of source contagiousness and of unobserved exposure. It was applied to data on influenza-like illness (ILI) among patients in a university hospital during 3 influenza seasons: from 2004 to 2007. Over a total of 138,411 patients-days of follow-up, 64 incident ILI cases were observed among 21,519 patients at risk of ILI.

**Results:**

The ILI risk per 10,000 patients-days associated with observed exposure was about 129.1 (95% Credible Interval (CrI): 84.5, 182.9) and was associated at 72% with exposures to patients or healthcare workers 1 day earlier and at 41% with the 1st day of source contagiousness. The ILI risk associated with unobserved exposure was 0.8 (95% CrI: 0.3, 1.6) per 10,000 patients-days in non-epidemic situation in the community and 4.3 (95% CrI: 0.4, 11.0) in epidemic situation.

**Conclusions:**

The model could be an interesting epidemiological tool to further assess the relative contributions of observed and unobserved exposures to CD risk in healthcare settings.

## Background

The occurrence of communicable diseases (CD) depends on exposure to contagious persons. This concept, called *dependent happenings*[1,2], should be considered in statistical models to assess CD risk, for example by incorporating explicative variables describing exposure [2].

Exposure to infection refers to the potentially infectious contacts a person has and some knowledge of natural disease history is needed to define exposure. Once a person exhibits symptoms of CD, only exposures that have occurred in period compatible with the incubation period of the susceptible host may lead to transmission. Also, exposure to an infectious individual could only lead to a subsequent infection (or disease) if exposure happens during the infectious period of the infected person. But, infectious periods and incubation periods differ individually and neither the durations of these periods nor the level of viral shedding are generally known. However, timing of exposure and contagiousness of the source are two important determinants of the risk of infection that can be included in statistical models aiming to analyze infectious diseases data.

Considering the moment of exposure, the effects of exposures to CD are often delayed in time and CD occurring at a given time can potentially be attributed to past exposures at different times (i.e. lags). The effects of exposure are distributed over a specific time period and can be modeled with several parameters to explain the contribution of exposures at different lags.

Similarly, regarding the source, contagious persons remain contagious for several days during which their contagiousness varies. It is, therefore, expected that the effects of exposure also vary with source contagiousness, the effects being distribution over the days of the contagious period.

Consequently, the effects of exposure on CD risk can be modeled as the joint result of delayed effects associated with lags and effects associated with the source person’s contagiousness, and a probabilistic approach may allow estimating the distributions of the risk according to lags and the source person’s contagiousness.

We propose a statistical model at the individual level to assess CD risk, in healthcare settings, among patients who may be subjected to multiple exposures. In this context, all sources of infection (patients, HCWs, or visitors) are usually imperfectly identified. So, it is important to consider not only observed exposure to other patients and healthcare workers (HCWs) but also exposures to unobserved or unobservable sources such as symptomatic infectious persons unnoticed during observation, asymptomatic persons or external sources. Incidence of CD in the community can be used to represent the unobserved exposures. The proposed model explores the delayed effect of multiple observed exposures, of source contagiousness and of unobserved or unobservable exposures. It was applied to data on influenza-like illness (ILI) among patients in a university hospital.

## Methods

### Model

A patient can contract a CD during his/her stay in a healthcare setting. This hospital-acquired CD may result from exposure to contagious HCWs or contagious patients as well as contagious persons coming from outside the healthcare setting, such as visitors. In addition, these exposures are usually imperfectly identified in epidemiologic studies because generally clinical cases are of interest. Exposures can be observed (i.e. documented), unobserved (i.e. unnoticed) or unobservable (e.g. asymptomatic contagious persons). In the following, only the term “unobserved exposure” will be used but it must be remembered that it referred to “unobserved and unobservable exposure”. Separating these two effects is out of the scope of the present work.

When a hospitalized person has a CD, multiple exposures delayed in time may be consistent with disease onset and it is difficult to know with certainty which exposure is associated with the CD. The model is based on the concept that escaping to infection from each exposure is required to avoid infection. If *λ*_*ij*_^*observed*^ is the CD risk associated with observed exposure (*w*_*ij*_), then the cumulative risk of escaping CD following a series of exposures is ${\prod_{\mathrm{ij}}\left(1-\lambda_{\mathrm{ij}}^{\mathrm{observed}}\right)}^{w\mathrm{ij}}$. In other words, to avoid CD, a patient should escape infection from all exposures.

To estimate the CD risk to patients, taking into account the effect of observed exposure associated with lags and the day of source person contagiousness as well as unobserved exposure, we constructed the model at the individual level, so that, for a given patient and day, CD risk λ can be modeled as follows:

$$\begin{array}{c} y∼\mathrm{Bernoulli}\;\left(\lambda \right)\\ \lambda =1-\left(1-\lambda^{\mathrm{unobserved}}\right)\prod_{\mathrm{ij}}{\left(1-\lambda_{\mathrm{ij}}^{\mathrm{observed}}\right)}^{w_{\mathrm{ij}}} \end{array}$$

where *y* = 1 if the patient has a CD and *y* = 0 if not, *w*_*ij*_ is observed exposure, equals 1 if the patient has been exposed to patients or HCWs *i* days earlier at their *j*^*th*^ contagiousness day and *w*_*ij*_ = 0 if not, *λ*_*ij*_^*observed*^ is the CD risk associated with observed exposure, and *λ*^*unobserved*^ is the CD risk associated with unobserved exposure. As represented by *y*, the model includes ILI cases and non-cases. Patients were followed since admission at hospital. Censoring occurred for cases at the time of ILI and non-cases were followed up to discharge. So each admitted patient contributed to the risk set, until onset of ILI for cases and until discharge for non-cases. In addition, ILI patients were assumed to be immunized after ILI and therefore no more at risk.

To define maximum lag, we assume a maximum incubation period of the CD of *I* days. Thus, exposure of a patient can be defined as the presence of a contagious person in the same ward during the preceding *I* days. Contagious persons remain contagious for a given period and in the following, we assume a maximum contagiousness period of *J* days.

#### Observed exposure

For capturing the effects of observed exposure, we used an approach belonging to the context of the transmission models based on “who acquires infection from whom” (WAIFW) matrices , for which estimation methods are available elsewhere [3,4]. The CD risk observed exposure can be decomposed as follows (see Additional file 1):

$$\lambda_{\mathrm{ij}}^{\mathrm{observed}}=1-\exp\left(-k\;a_{i}\;b_{j}\right)$$

with constraints $\sum_{i}a_{i}=1$ and $\sum_{j}b_{j}=1$, where *k* is a scale parameter representing the CD risk, [*a*_*1*_,…,*a*_*i*_,…,*a*_*I*_]^T^ being the distribution vector of this risk over the preceding *i* days, and [*b*_*1*_,…,*b*_*j*_,…,*b*_*J*_] being the distribution vector of risk over *j* days of source contagiousness. This decomposition in 2 dimensions allows easy interpretation of parameters and makes the assumption of independence between the effect of lags and of the day of contagiousness. It also supposes that the risk is constant a given day for a given individual which is plausible since exposure is not expected to vary in this interval.

#### Unobserved exposure

In the model, we allowed exposures to unobserved sources such as exposures to symptomatic contagious persons unnoticed during observation, asymptomatic persons or external sources. We further assumed that unobserved exposure depended on the community incidence of CD. The CD risk associated with unobserved exposure in a healthcare setting can then be written as follows:

$$\lambda^{\mathrm{unobserved}}=\exp\left(\beta^{\mathrm{Non}-\mathrm{epidemic}}+\beta^{\mathrm{Epidemic}}x\right)$$

where *x* = 1 if the epidemic threshold has been reached on a given day in the community and *x* = 0 if it has not been reached. When CD incidence in the community is below the epidemic threshold, *λ*^*unobserved*^ = exp(*β*^*Non* − *epidemic*^). This risk is increased by factor *β*^*Epidemic*^ when the incidence in the community is above the epidemic threshold.

### Inference methods

We combined two methods for inference. First, maximum likelihood estimates of the model parameters were estimated as well as their 95% confidence intervals (95% CI) obtained from the Hessian matrix. The optim function in R was used. Then, the number of cases being small, a Bayesian inference was performed to obtain more robust intervals estimates. MCMC (*Markov Chain Monte Carlo*) methods [5] gave posterior parameters means and 95% credible intervals (95% CrI) from 2.5% and 97.5% quantiles of posterior distribution. Parameters were assigned non-informative priors [6]. Three independent MCMC with 3 different initial values were run in parallel to assess convergence on posterior parameter distributions and to check that the results were not sensitive to the choice of starting values. Gelman and Rubin convergence criteria [7] was also calculated and examined. After an initial burn-in period of 20,000 iterations, 80,000 iterations served to compute posterior parameter distributions. MCMC were analyzed with OpenBugs and the coda package in R.

### Exposure to ILI in healthcare settings

The model was applied to assess the effects of exposure on ILI risk in healthcare settings. The data originated from 3 sources. The first source was a prospective observational study carried out between November 15, 2004 and April 15, 2007 at Edouard Herriot Hospital in Lyon, France [8]. During the study, each participating ward was followed-up daily to detect ILI cases. A case was defined as an adult patient or HCW presenting with fever (≥37.8°C) and cough or sore throat. A non-case was defined as a patient or a HCW free from ILI during the study period. As secondary source, all hospital data (admission/discharge dates and ward) on cases and non-cases were extracted from the hospital’s information system. Data on HCW work periods were included. To quantify exposure to ILI and to define lags, the maximum incubation period was set at *I* = 5 days [9]. Therefore, for each patient, exposure was defined as the presence of a contagious person in the same ward and on 5 previous days. We assumed a contagious period for ILI of *J* = 6 days, starting 1 day before symptom onset [9]. Having a small number of cases, exposures to contagious persons 4 and 5 days earlier were grouped together. Similarly, exposures occurring during the 4th to 6th days of the source contagiousness were pooled. The third source was the French Institute for Public Health Surveillance [10], for defining epidemic and non-epidemic periods in the Rhône-Alpes region.

We assumed that infected patients didn’t become susceptible again and therefore follow-up was censored the day of ILI onset for cases and the day of discharge for patients free from ILI. Over a total of 138,411 patient-days (19,773 weeks) of follow-up, 64 incident ILI cases were observed among 21,519 patients at risk of ILI.

## Results

Parameter estimation by maximum likelihood and Bayesian inference gave similar results (Table 1).

**Table 1:** Parameter estimates of Influenza-Like Illness (ILI) risk in a university hospital by 2 inference methods

	**Maximum likelihood**		**Bayesian inference**	
	**Mean**	**95% confidence interval**	**Mean**	**95% credible interval**
**ILI risk associated with unobserved exposure (per 10,000 patients-days)**				
No epidemic in the community	0.8	0.3-1.6	0.8	0.3-1.6
Epidemic in the community	4.3	0.4-11.0	4.3	0.4-11.0
**ILI risk associated with observed exposure (per 10,000 patients-days)**	129.1	84.5-182.9	129.1	84.5-182.9
**Distribution of risk according to lags**				
1 day	82	51-95	72	52-89
2 days	1	0-98	6	0-21
3 days	15	0-81	12	1-32
4 to 5 days	2	- *	10	0-27
Total	100	-	100	-
**Distribution of risk according to the source’s contagious period**				
1 day before the onset of source symptoms	47	24-71	41	21-62
1st day after the onset of source symptoms	17	6-42	19	4-39
2nd day after the onset of source symptoms	24	8-51	25	9-44
3rd to 5th days after the onset of source symptoms	12	- *	14	4-29
Total	100	-	100	-

*Confidence interval not computable because this parameter was inferred from others.

The daily ILI risk per 10,000 patients-days associated with observed exposure was 129.1 (95% CrI: 84.5, 182.9). The ILI risk associated with unobserved exposure was 0.8 (95% CrI: 0.3, 1.6) in non-epidemic situation in the community and 4.3 (95% CrI: 0.4, 11.0) in epidemic situation. The distribution of ILI risk according to lags was 72%, 6%, 12% and 10% for observed exposures occurring 1, 2, 3 and 4–5 days earlier respectively. The distribution of this risk was 41%, 19%, 25% and 14% during observed exposure to patients or HCWs 1 day before their symptom onset, 1st, 2nd and 3rd to 5th days after symptom onset respectively.

**Figure 1:**
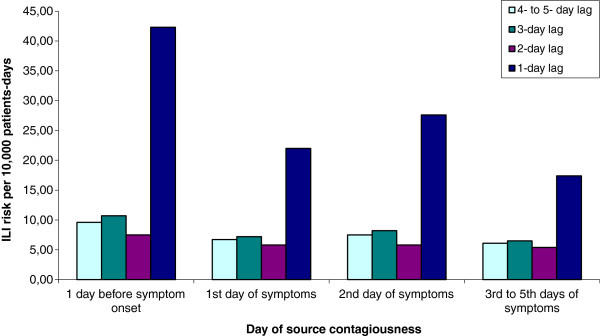
**Risk of Influenza-Like Illness (ILI) associated with exposure to a patient or a healthcare worker according to a given lag and day of source contagiousness in a healthcare setting during community epidemic period. **Each bar represents the risk of ILI associated with one exposure at a given lag and a given day of contagiousness, in the absence of all other exposures.

**Figure 2:**
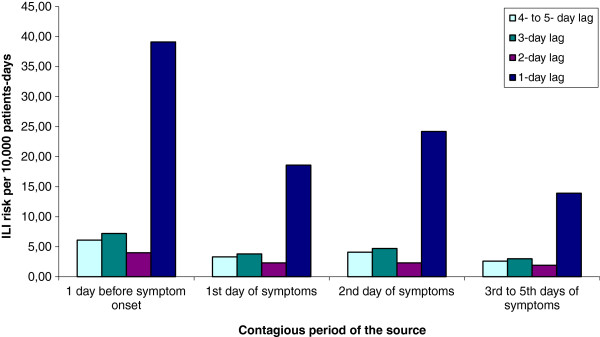
**Risk of Influenza-Like Illness (ILI) associated with exposure to a patient or a healthcare worker according to a given lag and day of source contagiousness in a healthcare setting outside community epidemic period. **Each bar represents the risk of ILI associated with one exposure at a given lag and a given day of contagiousness, in the absence of all other exposures.

Figures 1 and 2 present the daily ILI risk according to the day of contagiousness, lags and epidemic threshold in the community (see Additional file 1 for details of calculations). In the figures, each bar represents the risk of ILI associated with one exposure at a given lag and a given day of contagiousness, in the absence of all other exposures. The results indicate that ILI risk was highest when observed exposure took place the previous day whatever the day of contagiousness and ILI incidence in the community. A slight decrease in risk was observed with the day of contagiousness.

## Discussion

In the present work, a statistical individual model allows estimation of CD risk to patients who may have had multiple observed exposures and unobserved exposures in healthcare settings. The model is defined at the patient and daily levels, which allowed capturing individual and temporal variations of observed and unobserved exposures to finally precisely assess the relationship between exposures and CD risk. An additional feature of the model is to integrate the natural history of the disease. Then, the model can serve to identify days of exposure associated with increased risk of CD as well as days of contagiousness associated with heightened risk of disease transmission.

Application of this model to ILI showed that, on any given day, the observed ILI risk among adult patients was 129.1 per 10,000 patients-days and was mainly associated with exposures to patients or HCWs 1 day earlier and during the 1st day of source contagiousness. These results suggest 1) that the incubation period of ILI could be ≤24 h, and 2) that ILI could be transmitted when the source has no symptoms yet (for ILI, contagiousness starts 1 day before symptom onset [9]). In addition, the ILI risk appeared to decrease gradually, depending on the day of contagiousness. These results are consistent with current knowledge on ILI [9,11], which supports potential application of the model in hospital epidemiology.

These results indicate that prevention measures, such as closing wards, restricting visits, avoiding contact between healthy patients and contagious patients or HWCs, isolating contagious persons, or antiviral treatment and prophylaxis, should be started as soon as a case is suspected. A delay in alert or implementation of these measures could increase the risk of transmission. In addition, contact tracing may be useful to identify patients who may have been in contact with a contagious symptomatic patient or HCW the day preceding his or her symptom onset. These results are proposed for ILI and influenza among adult patients, but the model could be easily extended to children with longer contagious periods, to different subtypes of influenza viruses with various natural histories, and to other infections sharing similar routes of transmission and natural histories.

In comparison to observed exposure, unobserved exposure accounted for a small part of the total transmission. This may suggest that contagious persons unnoticed during observation, asymptomatic persons or external sources may play a limited role in ILI transmission in healthcare settings, compared to symptomatic sources. It is unlikely that symptomatic persons or external sources were missed because the ILI definition we used had a high sensitivity. A lower infectiousness of asymptomatic sources may also explain this slight effect. However, interestingly, the effect of unobserved exposure seems to increase, by a 5-fold factor, during epidemic period compared to non-epidemic period. In absence of an active surveillance, this result is in favor of limiting exposures during epidemic period or during emergencies such as a pandemic. It could be also an argument to promote influenza vaccination for patients as well as for HCWs.

The approach presented here had some limitations. Exposure was defined as the presence of a contagious person in the same ward and this definition ignored information on actual contacts between persons. In practice, however, it is difficult to follow each individual to find out with whom, when and for how long he/she came into contact with other individuals. In addition, the definition of exposure may seem more appropriate than contact because it includes all ILI transmission types. We did not follow patients outside hospital and it is possible that some patients presented ILI just after discharge. In this case, it is possible that transmission was underestimated. The bias was limited, the statistical model taking into account the censoring process, i.e. the discharge of the patients. The model was based on the assumption of independence between the lag of exposure and the day of source contagiousness. While this assumption appeared to be weak in our case, there were other ways to estimate the parameters of a WAIFW matrix, for example, by equality constraints between parameters [3]. However, this hypothesis had the advantage of facilitating the estimation and interpretation of parameters. Equality constraints seem to be not entirely appropriate and could limit study of the influence of the day of contagiousness and of the lag of exposures on CD risk. However, shape constraints [12], reflecting the natural history of disease or adding *a priori* information, should be considered in future improvements of the model. For example, incubation [9,11] and contagiousness periods [9] follow upswing and downswing trends, and it would be interesting to consider such information in the model.

Due to the hierarchical structure of the hospital (i.e. patients in wards and wards in the hospital), it is likely that patients in the same wards may be not independent. A mixed effects model would be an interesting extension of the model, by adding a ward-specific scale parameter (*k*) representing a possibly different CD risk for each participating ward in the study. However the low number of events per ward (some wards experienced no ILI) would not have permitted to obtain reasonable estimates under the mixed-effects version of the model. It would be relevant to apply this model and its extensions to larger datasets.

## Conclusions

Our results disclosed that the presence of a contagious patient or HCW dramatically increased the risk of ILI to other patients. Therefore, the proposed statistical model could be an interesting epidemiological tool to further assess the relative contribution of observed and unobserved exposures to CD risk in healthcare settings. Beyond the statistical approach, these results will be helpful to improve daily prevention of CDs in hospitals.

## Competing interests

The authors declare that they have no competing interests.

## Authors’ contributions

CP, NV and RE designed the model, performed statistical analyses and wrote the manuscript. PV provided access to the data and helped in the preparation of the manuscript. All authors approved the final submitted version.

## Pre-publication history

The pre-publication history for this paper can be accessed here:

http://www.biomedcentral.com/1471-2288/13/26/prepub

## Supplementary Material

Additional file 1 Decomposition of CD risk associated with observed exposure. Click here for file
