# Supplementary material for: A statistical model to assess the risk of communicable diseases associated with multiple exposures in healthcare settings
Source: BMC Med Res Methodol. 2013 Feb 20;13:26. doi: 10.1186/1471-2288-13-26 (PMC3599894; doi:10.1186/1471-2288-13-26)
Supplement: Additional file 1 — Decomposition of CD risk associated with observed exposure. [file 1471-2288-13-26-S1.doc]

## Additional file 1

**Decomposition of CD risk associated with observed exposure**

For a given patient and given day, the maximum periods of incubation and contagiousness are *I* and *J* days, respectively. For a given patient and day, CD risk *λ* is modeled by:

$$\lambda=1-(1-\lambda^{unobserved})\prod_{ij} (1-\lambda_{ij}^{observed})^{w_{ij}}$$

where *w_ij_* is observed exposure, equals 1 if the patient has been exposed to patients or HCWs *i* days earlier at their *j^th^* contagiousness day and *w_ij_* = 0 if not, $\lambda_{ij}^{observed}$ is the CD risk associated with observed exposure, and $\lambda^{unobserved}$ is the CD risk associated with unobserved exposure.

Then,

$$\lambda=1-\exp(\log(1-\lambda^{unobserved})+\sum_{ij} w_{ij}\log(1-\lambda_{ij}^{observed}))$$

And,

$$\lambda=1-\exp(\log(1-\lambda^{unobserved})+\sum_{ij} w_{ij}\beta_{ij})$$

with $\beta_{ij}=\log(1-\lambda_{ij}^{observed})$

For ease of interpretation, observed exposures *w_ij_* can be represented using an observed exposure matrix **W**:

Day of source contagiousness (*j*)

1 … *J*

$$\mathbf{W}=\left(\begin{matrix} w_{11} & ... & w_{1J} \\ ... & ... & ... \\ w_{I1} & ... & w_{IJ} \end{matrix}\right)$$

1

…

*I*

Lag (days, *i*)

Similarly, the effects *β_ij_* associated with exposures *w_ij_* can be represented using a matrix **β** (Note that **β** is a WAIFW type):

Day of source contagiousness (*j*)

1 … *J*

$$\mathbf{\beta}=\left(\begin{matrix} \beta_{11} & ... & \beta_{1J} \\ ... & ... & ... \\ \beta_{I1} & ... & \beta_{IJ} \end{matrix}\right)$$

1

…

*I*

Lag (days, *i*)

The matrix **β** can be further decomposed as follows:

$$\mathbf{\beta}=-k \mathbf{a}^{T}\mathbf{b}$$

with $\sum_{i} a_{i}=1$ and $\sum_{j} b_{j}=1$ and where *k* is a scale parameter representing CD risk on a given day, [*a_1_*,…,*a_i_*,…,*a_I_*] ^T^ being the distribution vector of this risk over preceding *I* days and [*b_1_*,…,*b_j_*,…,*b_J_*] being the distribution vector of risk over *J* days of source contagiousness. Decomposition in 2 dimensions makes the assumption of independence between the effect of the lag and of the day of contagiousness.

So for each element of **β**,

$$\beta_{ij}=\log(1-\lambda_{ij}^{observed})=- k a_{i} b_{j}$$

And,

$$\lambda_{ij}^{observed}=1-\exp(-k a_{i} b_{j})$$

Note that when CD risk is very low (<1%), the following approximation can be made:

$$\lambda_{ij}^{observed}\approx ka_{i}b_{j}$$

This decomposition in 2 dimensions allows easy interpretation of parameters and the model can also be written as:

$$\lambda=1-\exp(\log(1-\lambda^{unobserved})-k\mathbf{aWb}^{T})$$

**Calculations for Figures 1 and 2**

In the tables, each cell represents the risk of ILI associated with one exposure at a given lag and a given day of contagiousness, in the absence of all other exposures. For example, in Table 1, λ=5.7 was obtained using parameters estimates and $\mathbf{W}=\left(\begin{matrix} 0 & 0 & 0 & 0 \\ 0 & 1 & 0 & 0 \\ 0 & 0 & 0 & 0 \\ 0 & 0 & 0 & 0 \end{matrix}\right)$. These tables were used for constructing Figures 1 and 2.

**Additional file Table 1.** ILI Risk per 10,000 Patients-Days in a Healthcare Setting According to the Source’s Contagiousness day and the lag in an Epidemic Situation (Values Corresponding to Figure 1)

|  | Epidemic in the community | | | | |
| --- | --- | --- | --- | --- | --- |
|  | Day of source contagiousness | | | | |
| Lag |  | 1 day before symptom onset | 1^st^ day of symptoms | 2^nd^ day of symptoms | 3^rd^ to 5^th^ days of symptoms |
|  | 1 day | 42.3 | 22.0 | 27.6 | 17.4 |
|  | 2 days | 7.5 | 5.7 | 5.8 | 5.4 |
|  | 3 days | 10.7 | 7.2 | 8.2 | 6.4 |
|  | 4 to 5 days | 9.6 | 6.7 | 7.5 | 6.1 |

**Additional file Table 2.** ILI Risk per 10,000 Patients-Days in a Healthcare Setting According to the day of Source Contagiousness and the lag in a non-Epidemic Situation (Values Corresponding to Figure 2)

|  | No epidemic in the community | | | | |
| --- | --- | --- | --- | --- | --- |
|  | Day of source contagiousness | | | | |
| Lag |  | 1 day before  symtom onset | 1^st^ day of symptoms | 2^nd^ day of symptoms | 3^rd^ to 5^th^ days of symptoms |
|  | 1 day | 39.1 | 18.6 | 24.2 | 13.9 |
|  | 2 days | 4.0 | 2.3 | 2.3 | 1.9 |
|  | 3 days | 7.2 | 3.8 | 4.7 | 3.0 |
|  | 4 to 5 days | 6.1 | 3.3 | 4.1 | 2.6 |
